# The epidemiology and management of tibia and fibula fractures at Kilimanjaro Christian Medical Centre (KCMC) in Northern Tanzania

**DOI:** 10.11604/pamj.2016.25.51.10612

**Published:** 2016-09-29

**Authors:** Samuel John Clelland, Priyesh Chauhan, Faiton Ndesanjo Mandari

**Affiliations:** 1 College of Medical and Dental Sciences, University of Birmingham, Edgbaston, Birmingham, B15 2TT, United Kingdom; 2 Kilimanjaro Christian Medical Centre, PO Box 3010, Moshi, Tanzania

**Keywords:** Epidemiology, tibia, fibula, fractures, Tanzania, orthopaedics

## Abstract

**Introduction:**

Tibia/fibula fractures are one of the commonest admissions to the orthopaedic department at a resource-limited Northern Tanzanian hospital. These fractures are associated with poor prognosis and pose a huge socioeconomic burden on developing countries. However, to date there is a paucity of epidemiological data on lower-limb fractures in Tanzania.

**Methods:**

A retrospective review of admissions to the orthopaedic department at Kilimanjaro Christian Medical Centre (KCMC) was completed between February 2015 and 2016. Inpatient record books were used to source epidemiological data which was subsequently analysed.

**Results:**

199 of the 1016 patients admitted sustained tibia/fibula fractures. 78% (n=156) of admissions were male and the most frequently affected age group was 21-30 years. Motor traffic accidents (MTAs) were the most common cause and accounted for 78% of fractures, with nearly half of these involving motorbikes (42%). Falls were identified as the second most common cause (13%). It was determined that 72% (n=143) of fractures were open, 19% (n=38) were comminuted and the most common site of injury was the distal-third of tibia/fibula. The most frequently recorded treatments were surgical toilet/debridement (66% of patients) and the application of a backslab (34% of patients).

**Conclusion:**

Males in the 21-30 age group, who were involved in MTAs, were most commonly affected by tibia/fibula fractures. Given that MTA incidence is increasing in Tanzania, there is a growing public health concern that this will be reflected by a step-increase in the number of people who sustain lower-limb fractures.

## Introduction

Motor traffic accidents (MTAs) are associated with significant morbidity and mortality in low-income countries such as Tanzania [1]. It has also been reported by Chalya et al. that a large proportion of road traffic collisions result in fractures of the lower-limb [1]. At Kilimanjaro Christian Medical Centre (KCMC) open fractures of the femur, tibia and fibula are recognised as having a particularly poor prognosis [2]. In 2014, open lower-limb fractures were identified as the second most common cause of death in the hospital's orthopaedic department [2]. This statistic reiterates the devastating health implications associated with this particular type of injury. It is well-documented that tibia fractures are the most common type of long bone fracture in the developed world [3].

However, given that there is no established culture for routine data collection in East Africa, there is an apparent lack of available literature which specifically investigates the incidence, epidemiology, aetiology and management of tibia/fibula fractures in low-income countries such as Tanzania. This has been highlighted previously by Hollis et al. [4], who considered the epidemiology and management of femoral fractures at KCMC. In recent years, several studies have demonstrated a marked increase in the number of MTAs in low- and mid-income countries [5]. It is interesting to note that within these countries there has also been a proportional increase in the number of motor vehicles and the levels of urbanisation [6]. Developing countries, such as Tanzania, account for more than 85% of all MTA deaths worldwide; they are also associated with a disproportionately high number of deaths and disability compared to the developed world [7]. Open tibia fractures are commonly caused by high-impact trauma, for example as a result of an MTA.

In general, MTAs represent a well-documented growing disease burden in the developing world and therefore pose a significant cause for concern [8]. Given the lack of infrastructure to facilitate the proper reporting and documentation of MTAs in the developing world, road traffic injuries and fatalities are significantly under-reported [9]. The World Health Organization (WHO) road safety statistics for Tanzania indicate that in 2013, over 4000 people were reported to have died as a direct result of an MTA. However, they estimate that the actual figure is more likely to be closer to 16000 fatalities [10]. The substantial socioeconomic burden of MTAs is clearly demonstrated in the 2007 WHO road safety statistics, which estimate that Tanzania lost 3.4% of their gross domestic product as a direct result of road traffic collisions [11]. It has been predicted by 2020 that MTAs will be the 6th largest cause of death and 3rd largest cause of disability in Tanzania [12]. There is therefore an urgent need for appropriate public health interventions to be put in place to curb this trend. Whilst literature relevant to fracture epidemiology and management is scarce in Tanzania, some useful studies have been undertaken in recent years. Casey et al. [8] performed a retrospective analysis of patients presenting to the emergency department at KCMC and found that MTAs were the most common cause of injury (43% of all admissions). Extremity injuries, including tibia/fibula fractures, were also noted to be extremely common (59.5% of all admissions). Casey et al. [8] highlighted the need for further studies which specifically investigate the mechanism of injury and the type of injury sustained in MTAs. This gap in the literature is an aspect which we aimed to address in our epidemiological study. Hollis et al. [4] performed a retrospective descriptive study of the epidemiology of femoral fractures. They concluded that MTAs were the most common cause of femur fractures and that they most frequently affected males under the age of 30. However, this study did not evaluate tibia or fibula fractures. Chalya et al. [1] and Schaftenaar et al. [13] have also both performed epidemiological research in Tanzania but only looked at MTA injuries and maxillofacial trauma patients, respectively. At present, there is no available literature investigating tibia/fibula fractures as a primary outcome despite the significant morbidity, mortality and economic burden associated with lower-limb fractures. The aim of this retrospective, descriptive study is to identify the epidemiology, aetiology, type and management of tibia and fibula fractures in patients admitted to an orthopaedic ward at KCMC.

## Methods

The study was undertaken in the orthopaedic department at KCMC, a large tertiary referral centre in North Tanzania. KCMC is the country's third largest hospital serving a catchment area of over 15 million people [2]. The orthopaedic department was founded in 1986 and consists of 70 inpatient beds spread over six general and four private bays. The University of Birmingham and the head of the orthopaedic department at KCMC both gave approval to carry out this study. Inpatient orthopaedic admission record books were sourced from February 2015 to February 2016 and were retrospectively reviewed, forming a 12-month dataset. Between the dates of 10/09/2015 and 29/10/2015, documentation in the inpatient record books was untraceable. During this time period, nursing admission books were used to source a more restricted dataset, which was limited to age, gender, type of fracture and associated fractures. All tibia and fibula fractures within the specified 12-month time period were included in the study; with the exception of malleolar fractures which were excluded as they were judged to be fractures of the ankle joint. To minimise the potential for inappropriate inclusions or exclusions from the study, all data sourced was obtained and cross-referenced independently by two reviewers. Information obtained from the orthopaedic admissions books included age, gender, pattern of fracture, other associated fractures, aetiology and the agreed management. The clerking intern obtained and documented this information for all patients upon admission to the orthopaedic ward. The aetiology was determined from the documented clinical history and the diagnosis from X-ray findings. Radiological imaging results were confirmed by a senior orthopaedic surgeon. Following data collection, the authors performed data analysis to establish associations between gender, age groups, pattern of fracture and mechanism of injury.

## Results

**Figure 1:**
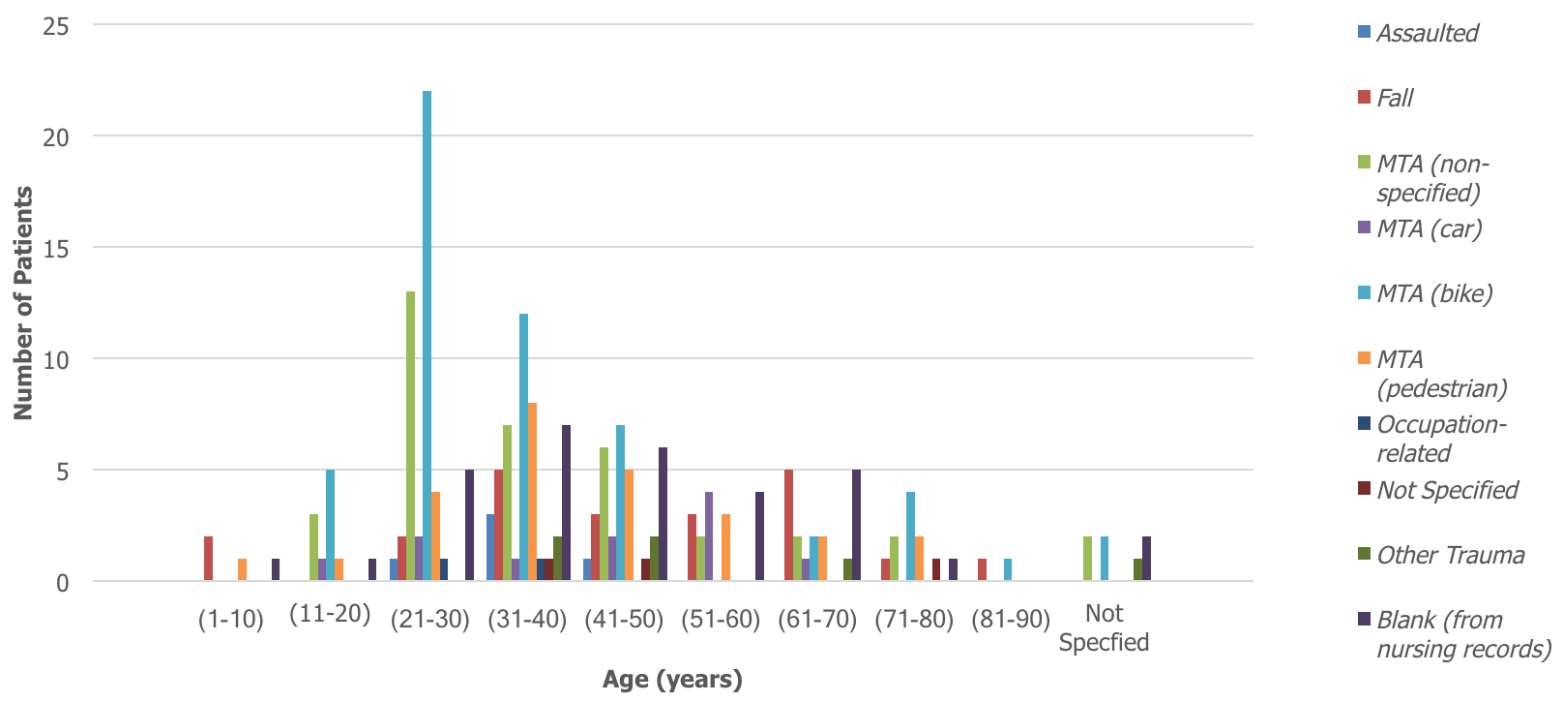
Fracture aetiology by age

**Figure 2:**
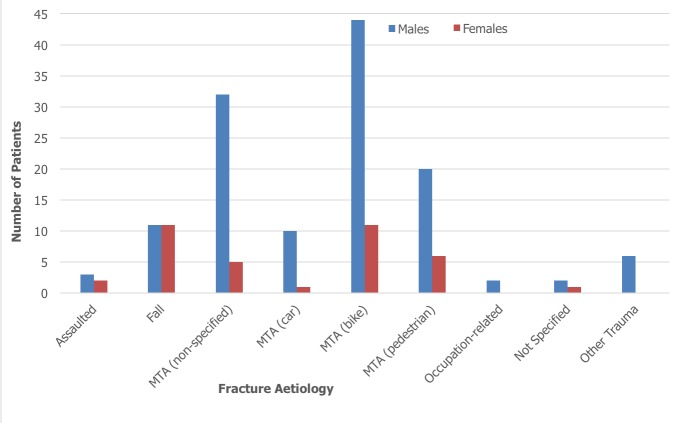
Fracture aetiology by gender

**Figure 3:**
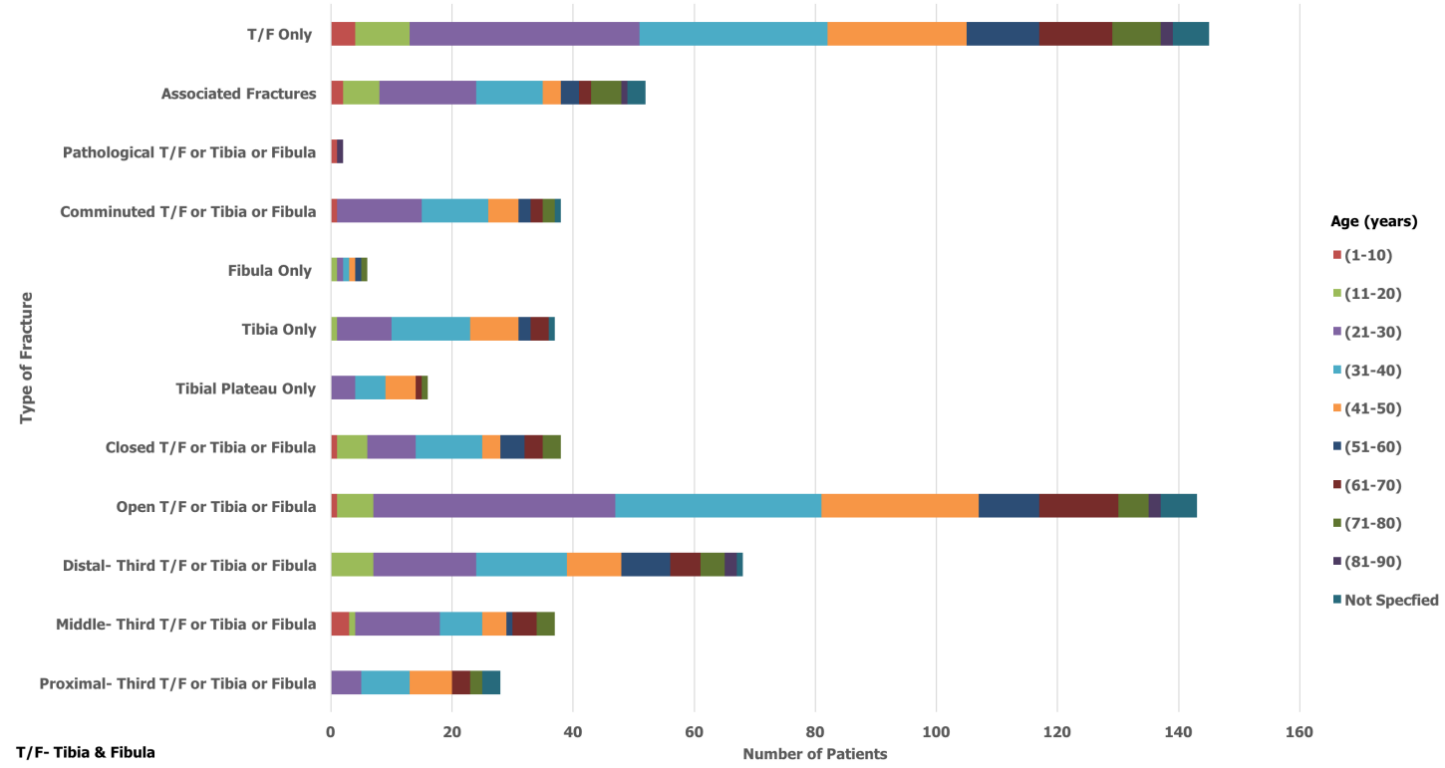
Fracture pattern by age

**Figure 4:**
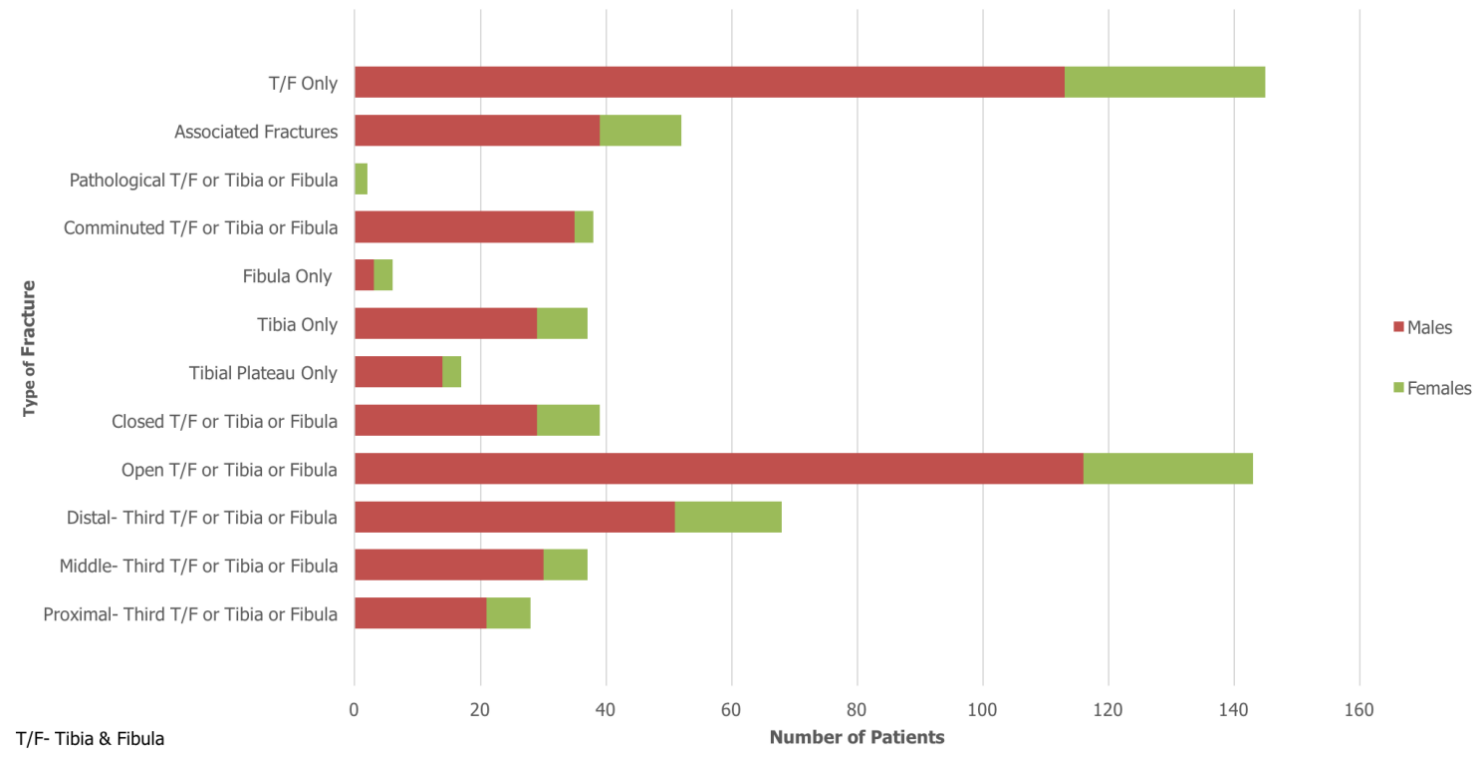
Fracture pattern by gender

During the 12-month period from February 2015 to 2016, a total of 1016 patients were admitted as inpatients to the orthopaedic department at KCMC. 199 out of 1016 patients sustained a tibia and/or fibula fracture, which equated to 20% of all admissions. 19% (n=37) of these patients sustained an isolated fracture of the tibia, 3% (n=6) sustained an isolated fracture of the fibula, with the remaining 78% (n=156) fracturing both bones. Of the 199 patients admitted, 156 (78%) were male and 43 (22%) were female. The average age of patients included in the study was 40 years with an age range of between 2-90 years. The most common age group affected was 21-30 years which made up 25% (n=50) of the total cohort. For males, the most common age group affected was 21-30 years which accounted for 31% (n=48) of fractures in males. For females, the most common age groups were 51-60 and 61-70 years which each accounted for 19% (n=8) of fractures in females. 167 of the available inpatient records specified aetiology of fracture. MTAs were by far the most common cause and accounted for 78% of tibia/fibula fractures. Falls were identified as being the second most common cause (13%, n=22). Of those who were involved in an MTA, 42% (n=84) were riding motorbikes, 9% (n=18) were driving cars, 21% (n=42) were pedestrians and 28% (n=56) were unspecified. For males, the most common cause of fracture was MTA involving a motorbike which resulted in 34% (n=44) of fractures. However, for females it was split equally between falls and MTAs involving a motorbike, which each accounted for 30% (n=11) of fractures. Figure 1 and Figure 2 summarise fracture aetiology according to age and gender respectively. When considering the pattern of fracture, it was found that 72% (n=143) of tibia/fibula fractures were open, 20% (n=39) were closed and 8% (n=17) were not specified. With regards to fracture location, 14% (n=28) of fractures were classified as involving the proximal-third, 19% (n=37) involved the middle-third and 34% (n=68) involved the distal-third. 9% (n=17) of fractures involved the tibial plateau whilst 19% (n=38) of fractures were described radiologically as comminuted. 26% (n=52) of patients sustained another fracture at a different site to that of the primary tibia/fibula fracture. Within this group, 75% (n=39) were male and 87% (n=45) sustained their injury though an MTA. Male patients (n=116) also sustained over four times as many open fractures as female patients (n=27). Figure 3 and Figure 4 summarise fracture pattern according to age and gender respectively. Overall, 66% (n=111) of patients were managed with surgical toilet/debridement and 34% (n=56) were managed with a backslab. It must be highlighted that data for 32 patients (16% of the total cohort) were sourced from the nursing admissions books. The agreed treatment for these patients was not recorded and subsequently could not be commented on or used in the analysis. 13% (n=21) of patients received a course of antibiotics, which usually consisted of a combination of ceftriaxone and gentamicin. The specific dosages, frequency and course length of the prescribed antibiotics were seldom recorded in the inpatient admission records. Only a small minority of patients underwent limb amputation (1%, n=2) following their tibia/fibula fracture. For 13% (n=21) of patients, the agreed treatment was not specified and for another 13% (n=22) external fixation was applied. 14% (n=24) of patients were treated with skeletal traction and 3% (n=5) of the cohort were managed with intramedullary nailing of the tibial shaft.

## Discussion

The aim of this retrospective descriptive study was to investigate the epidemiology and management of tibia and fibula fractures at a resource-limited Northern Tanzanian hospital. Our study found that males were approximately four times more likely to sustain a fracture of the tibia/fibula than females. The most common age group affected was 21-30 years which accounted for 25% of all fractures. The results collated in this study are also consistent with findings published by Twagirayezu et al. [14] and Chalya et al. [1] who identified that young males in particular were more likely to be involved in MTAs. Chalya et al. [1] analysed over 1600 MTAs in Tanzania and found that the ratio of males to females involved was approximately 2:1 and the overall modal age group was 21-30 years. A potential explanation for the fact that young males are more commonly involved in MTAs is that this subgroup usually pursues more dangerous occupations and undertakes higher-risk activities such as speeding, motorbike use, reckless driving and driving under the influence of alcohol [15]. Previous studies have also found that a large proportion of MTA victims were young businessmen or students [1]. As our study was not able to evaluate patient occupation, this may have been an important confounding variable influencing our results. Our study concluded that MTAs were the most common cause of tibia/fibula fractures. Of the 78% of fractures caused directly by involvement in an MTA, 42% involved motorbikes, 9% involved cars and 21% involved pedestrians. Again our results are in keeping with existing literature which has previously demonstrated that approximately 71% of femoral fractures involved motorbike-related accidents in Tanzania [4]. During the period of 1990 to 2000, the number of MTA-related deaths rose markedly in parts of the developing world, including in Tanzania which saw a 64% increase in MTA deaths [5]. According to the latest WHO statistics, it has been reported that a further 60% increase was seen in the 10-year period from 2004 (2500 deaths)-2014 (4000 deaths) [11]. However, it is likely that these figures are gross underestimates due to significant under-reporting of MTAs [11]. It has been suggested that there are several reasons for the dramatic rise in mortality rates including an increase in levels of urbanisation, poor road quality, reckless driving, poor law enforcement/regulation of road safety and the use of alcohol/recreational drugs whilst driving [5]. Increasing numbers of motorbikes on the road, in particular motorbike-taxi drivers, as well as an absence of valid driving licenses and safety equipment for these vehicles, has also been thought to have contributed to the changes seen in mortality rates [16]. Interestingly, Boniface et al. [17] found that more than 20% of patients involved in MTAs in Tanzania were suspected of driving under the influence of alcohol and less than 25% of MTAs involving motorbikes wore a helmet [1]. These statistics further highlight the urgent need for suitable public health initiatives to be implemented to ensure that drivers in developing countries are fully educated on all aspects of road safety and that proper road traffic laws are enforced. Due to socioeconomic differences, the aetiology of tibia/fibula fractures differs significantly in developing countries, such as Tanzania, compared to parts of the developed world. An epidemiological study [3] conducted in the UK over 3 years concluded that only 37.5% of fractures involved an MTA. This is clearly considerably less than the 78% reported in our study. Court-Brown et al. [3] also found that a larger proportion of fractures were caused by falls (27%) and that 31% of fractures occurred whilst participating in sporting activities. Interestingly, none of the patients who sustained a fracture of the tibia/fibula at KCMC did so through sports-related injuries.

The most common initial treatment documented in the inpatient records was surgical toilet/debridement which was used in 66% of patients. The second most common treatment was the application of a backslab which was used in 34% of patients. Only 3% of patients were treated with an intramedullary nail whilst external fixation was applied to 13% of patients. Current research has demonstrated that intramedullary nails are superior to both plate fixation and external fixation for open fractures [18]. The reasons behind the relatively low usage rates of intramedullary nails at KCMC are not fully established; however, it is suspected that the following are contributing factors: • Improper documentation of the complete management plan in the inpatient records. • A lack of appropriate surgical equipment due to the fact that intramedullary nails are imported from abroad. • An increased risk of infection if intramedullary nails are used as the first-line management option for patients presenting with open fractures. Certainly, a combination of these factors may explain why skeletal traction was used more frequently (14% of patients). It should also be noted that Surgical Implant Generation Network (SIGN) nails are becoming increasingly popular in the developing world. This is because they can be inserted without the need for expensive real-time radiological imaging, which is a technology that is currently inaccessible to most low-income countries. Our study found that 13% of patients received a combination of the antibiotics gentamicin and ceftriaxone. Optimal antimicrobial therapy, through microscopy, culture and sensitivities, was not achieved because of a lack of appropriate access to microbiology laboratories and because of financial constraints. Inconsistencies in treatment protocols and the fact that such a wide range of initial treatments were offered, highlights an urgent need for comprehensive, standardised national guidelines to be implemented to improve the quality of patient care. There are several limitations to this study. It should be noted firstly that as with all retrospective data collection studies, the quality of the results and conclusions drawn from it depend hugely on the accuracy and accessibility of the patient records. Some data from the inpatient admission books was incomplete, missing or of poor quality. For example, seven patient records failed to document age, three failed to document mechanism of injury and 21 failed to document the agreed treatment. As mentioned previously, no inpatient records were sourced between the dates 10/9/2015 and 29/10/2015; during this period the nursing admission books were used, however these contained a more limited data set. It is evident that for further epidemiological studies to be carried out more completely, a system of comprehensive record-keeping needs to be put in place at KCMC.

For retrospective analyses of this sort, it should be highlighted that there is an inherent risk of selection bias because the reviewers are not blinded in the study. However, in our case, this was overcome by selecting all tibia/fibula fractures in a predetermined time period with predetermined exclusion criteria. Data extraction and analysis was also performed independently by two separate reviewers. Given the lack of available data that we could feasibly source through the inpatient records, it was not possible to investigate potential confounding variables such as occupation or socioeconomic status. In addition, patient follow-up was not documented routinely and it was therefore not possible to assess this in our study. The authors were unable to investigate which treatments were subsequently offered by the orthopaedic team, after the initial treatment was administered. Important long-term clinical outcomes such as disability, mortality and quality of life post-discharge were not assessed. This clearly highlights the need for further prospective research to be carried out at KCMC. It is also important to note that the results collected in our study do not reflect the overall incidence of tibia/fibula fractures at KCMC as some simple closed fractures were discharged directly from the emergency department, with only outpatient follow-up. Consequently, the data collected by the authors will theoretically contain a larger proportion of complex, open and comminuted fractures. One final limitation that the authors acknowledge in this study was that the data collection was only performed in one location in Northern Tanzania; hence the conclusions drawn from our study may not be generalisable to all low-income African countries. Further research is therefore warranted to assess whether our conclusions are applicable to other countries in East Africa. The WHO has focused much of its efforts in recent years on public health initiatives to try and combat the increasing prevalence of MTAs in developing countries. However, there is clearly considerable work still to be done as over 85% of MTAs occur in low to mid-income countries [19]. There is an urgent need for primary prevention strategies to be implemented in order to educate the public on road safety, to develop new legislation and to enforce existing road traffic laws. This is because over 65% of MTAs in Tanzania were perceived to be due to dangerous or reckless driving [17]. There is also a need for improved vehicle maintenance and increased road side testing for drivers who are suspected to be driving under the influence of alcohol or recreational drugs. Given the limited treatment options available to patients who sustain a fracture, further investment is required to improve hospital facilities, access to clinical equipment and the expertise of the clinicians involved in clinical research and patient care.

## Conclusion

Young males between the ages of 21-30 years were the most commonly affected group of patients. MTAs, specifically involving motorbikes, were the most common cause of tibia/fibula fractures. Surgical toilet and the application of a backslab were the most common initial treatments. Given that such a wide range of treatment options are offered at KCMC, it is clear that comprehensive national guidelines need to be implemented in order to standardise patient care. The epidemiological data collected on tibia/fibula fractures in our study is useful in helping to gain insight into the limitations of healthcare provision in Tanzania, at both a local and national level. The authors anticipate that this study will be useful in helping to shape future public health policies and interventions. Further prospective research is required to investigate clinical outcomes such as the duration of hospital admissions, levels of disability and mortality rates. Whilst time consuming, undertaking prospective research will enable a thorough assessment of potential confounding factors, such as occupation, financial income and associated co-morbidities, to take place. Additional research is required to fully evaluate and quantify the socioeconomic impact of tibia/fibula fractures in Tanzania. This will help to highlight disease burden and will also inevitably lead to increased investment into primary prevention strategies and treatment options.

### What is known about this topic

- Lower-limb fractures are associated with significant morbidity and mortality in low-income countries, such as Tanzania, and pose a huge socioeconomic burden.
- Several studies have demonstrated a marked increase in the incidence of tibia/fibula fractures in low and mid-income countries. This is thought to be as a direct result of an increase in the number of MTAs.
- There is a paucity of available literature which specifically investigates the incidence, epidemiology, aetiology and management of tibia/fibula fractures in Tanzania.

### What this study adds

- It summarises the epidemiology of tibia/fibula fractures in Northern Tanzania.
- It highlights the aetiology of tibia/fibula fractures in Northern Tanzania.
- It summarises the type of tibia/fibula fractures that present to the orthopaedic department at KCMC and how these fractures are subsequently managed.
